# Low-Cost Tracking Systems Allow Fine Biomechanical Evaluation of Upper-Limb Daily-Life Gestures in Healthy People and Post-Stroke Patients

**DOI:** 10.3390/s19051224

**Published:** 2019-03-11

**Authors:** Alessandro Scano, Franco Molteni, Lorenzo Molinari Tosatti

**Affiliations:** 1 Institute of Intelligent Industrial Technologies and Systems for Advanced Manufacturing (STIIMA), National Research Council of Italy (CNR), Via Alfonso Corti 12, 20133 Milan, Italy; lorenzo.molinaritosatti@stiima.cnr.it; 2 Villa Beretta Rehabilitation Center, Valduce Hospital, Via Nazario Sauro 17, 23845 Costa Masnaga (LC), Italy; fmolteni@valduce.it

**Keywords:** reaching, Kinect V2, biomechanical evaluation, upper-limb, post-stroke patients

## Abstract

Since the release of the first Kinect in 2011, low-cost technologies for upper-limb evaluation has been employed frequently for rehabilitation purposes. However, a limited number of studies have assessed the potential of the Kinect V2 for motor evaluations. In this paper, a simple biomechanical protocol has been developed, in order to assess the performances of healthy people and patients, during daily-life reaching movements, with focus on some of the patients’ common compensatory strategies. The assessment considers shoulder range of motion, elbow range of motion, trunk compensatory strategies, and movement smoothness. Seventy-seven healthy people and twenty post-stroke patients participated to test the biomechanical assessment. The testing protocol included four different experimental conditions: (1) dominant limb and (2) non-dominant limb of 77 healthy people, and (3) the more impaired limb of 20 post-stroke hemiparetic patients, and (4) the less-impaired limb of 11 patients (subgroup of the original 20). Biomechanical performances of the four groups were compared. Results showed that the dominant and non-dominant limbs of healthy people had comparable performances (*p* > 0.05). On the contrary, condition (3) showed statistically significant differences between the healthy dominant/non-dominant limb and the less-affected limb in hemiparetic patients, for all parameters of assessment (*p* < 0.001). In some cases, the less-affected limb of the patients also showed statistical differences (*p* < 0.05), with respect to the healthy people. Such results suggest that Kinect V2 has the potential for being employed at home, laboratory or clinical environment, for the evaluation of patients’ motor performances.

## 1. Introduction

The evaluation of motor performances of neurological patients is a common practice in clinical environment [1]. In fact, evaluations are at the basis of a correct selection of therapies to be administered, and are needed for measuring their effect. In a clinical environment, the standard tools for the assessments are clinical scales. Clinical scales are surveys and questionnaires that associate a score to specific performances, related to motor and cognitive aspects [2,3]. Despite providing a wide variety of assessments, clinical scales are inter- and extra-operator dependent, have intrinsic low sensibility, and suffer of ceiling and floor effects [4,5].

A deeper and quantitative assessment can be achieved with motion analysis or wearable sensors, which are some of the main techniques used in clinics to assess the motor capabilities of neurological patients. The clinical status and the effects of therapies can be evaluated in terms of motor performances, relative to the articular range of motion or motor control effectiveness [6,7,8,9]. Often, these analysis techniques can be coupled with biomechanical models, with the aim of increasing the knowledge about the disease [10]. The high precision of such methods, however, is associated with the fact that they require time-consuming acquisition and marker-positioning procedures, and cannot be used outside of the clinical environment.

There are evidences that the motor benefits obtained with rehabilitation are preserved, only if the motor function is frequently used in real life; the motor skill is more easily re-mastered if it is task-oriented and is performed with high intensity [11]. Thus, rehabilitation and motor monitoring should continue, even after de-hospitalization, in order to promote further motor improvement and use in daily life activities. In fact, according to the Schema Theory of motor learning [12], used in the literature to describe the motor learning process, both in physiology and pathology, brain-stored dynamical models underlying movement (motor programs) are mastered and refined if they are continuously trained.

At the same time, sanitary costs associated with clinical neurological rehabilitation and evaluation exams are growing consistently, and instruments capable of supervising rehabilitation and home training, acquire valuable and strategic potential, in particular the ones that allow motion tracking. In this scenario, the Kinect found fertile ground for exploitation, in rehabilitation. According to a recent study, several training paradigms have been considered for home-training, such as coaching, relying on intensity of repetition of specific motor gestures, biomechanical analysis and evaluation and feedback of the quality of motion, and gaming-based training, which instead enhances motivation and participation [13]. Exploitation of the Kinect technology included various applications, showing a high versatility [14], such as, assistance for motor gestures of children, or neurological patients [15,16], evaluation of cognition during training [17], support for serious games oriented to neuro-rehabilitation [18], supporting rehabilitative sessions during the execution of functional motor tasks connected to virtual reality environments, according to a gaming paradigm [19,20,21,22]. Fewer studies have evaluated the motor performances [23,24], confirming coherency with the results obtained with marker-based systems and clinical scales evaluations. In recent reviews, it was concluded that Kinect could be considered to be an adequate tool for supporting rehabilitation, and that expected future studies with Kinect, for rehabilitation applications, are extensive [25,26]. A lot of effort has been dedicated to discuss the affordability of Kinect, with respect to marker-based systems. Despite some differences in the range of motion (ROM) of body articulations, such as shoulder, elbow, hip, and knee, reproducibility of Kinect recordings, is comparable to marker-based systems [23,24,25,26,27]. This feature is especially interesting when considering biomechanical evaluations, since pre-post assessments must be reliable, repeatable, and comparable, even if there is some bias, with respect to the marker-based systems. It was also found that the Kinect-based 3D reachable workspace analysis of the upper-limb, provides sufficiently accurate and reliable results, as compared to the motion capture systems [28]; similar results were also found when evaluating shoulder ROM [29]. On the contrary, a study instead reported consistent discrepancies in evaluating shoulder angles [30], even if such results are coupled with a good repeatability of the measures. 

The release of Kinect V2, led to applications based on interactions with simple virtual environments or games that couple functional rehabilitation with gaming approaches [31]. Kinect V2 was chosen for a wide range of applications, including evaluations of the motor performance to provide clinicians with quantitative assessment [32], gait analysis [33], and head pose estimation [34]. Despite the increased accuracy and tracking algorithms, a limited number of studies reported the use of the Kinect V2 sensor as an instrument for providing evaluations of the biomechanics of the movement and the quality of motor control of the upper-limb. Articular range of motion was evaluated, concluding that Kinect V2 precision is acceptable for clinical applications or evaluation of motor performances of the upper-limb [35]. Kinect V2 was proposed as a tool for evaluating the biomechanics of the upper-limb [36], indicating, with preliminary results, that Kinect V2 might be suitable for patient evaluation, by providing a coherent assessment with respect to clinical scales. However, as previously mentioned, a reduced number of studies evaluating Kinect V2 performances on patients, in comparison to a golden standard marker-based systems method was found. In their work, Otte et al. [37] found an excellent agreement between the Kinect and Vicon gold standard, as well as retest reliability for a variety of kinematic parameters extracted from different motor tasks of clinical interest, on a wide sample of neurological patients [37]. In another study [38], a detailed analysis was performed to verify the suitability of Kinect V2 as a tool to evaluate rehabilitation of the upper-limb, coming to the encouraging conclusion that the device is suitable for rehabilitation applications. However, it should be underlined that several studies have indicated the adequacy of the sensor on healthy people tracking, aimed at motor evaluations, suggesting its application to pathological movement. In fact, comparing Kinect V2 and a marker-based system, in Otte et al. [37], it was found that, in summary, most clinical parameters showed a high absolute agreement and no systematic bias between systems, and that the parameters that showed moderate absolute agreement, mostly showed a high consistency in agreement, as well. Similar results were found in the Parkinson Disease Assessment [39], in gait analysis and evaluation [40], and for dynamic movements in rehabilitation scenarios [41]. A small cohort of neurological patients was clinically evaluated by the means of Kinect V2 [42]. A recent study [43] assessed Kinect V2 as a tool for evaluating spinal muscular atrophy patients, matched with healthy controls, concluding that the Kinect V2 sensor had the potential of being developed into a complementary output measure, as it provided reproducible, objective, and detailed information of body point motion. In addition, in the issue of Kinect V2 repeatability, with promising results that were to be further investigated, was addressed in a recent study [43]. Kinect V2 was used to record the kinematics of the upper-limb as trigger for functional electrical stimulation of a robotic set-up, aimed at assistance in home environment, confirming a high confidence on the system reliability [44]. Furthermore, a detailed study, not oriented to rehabilitation, underlined that Kinect V2 performances were evidently higher than the Kinect ones [45]. Embedded algorithms for joint tracking made it one of the most valuable, despite affordable, substitute of marker-based systems.

Last, there is a growing literature that works at merging clinical scales and low-cost tracking devices. In fact, the recent work of some authors, was aimed at automatizing some clinical, commonly used assessments, such as proximal arm non-use (PANU) [46,47], the Fugl Meyer Assessment [48], or the Reaching Performance Scale [49]. This approach seemed a natural and valuable use of the device, exploiting its ease-of-use, to standardize the already existing clinical exams.

However, only a reduced number of studies have assessed in fine details, the potential of the system in discriminating healthy people and pathology, and in comparing performances between healthy people and neurological people performances, especially in a context of biomechanical evaluations, rather than in a training platform test.

In the framework of a group of Research Projects in Northern Italy aiming at developing technologies for the improvement and evaluation of patients with motor disabilities, a simple but consistent upper-limb functionality evaluation module, coupled with low-cost technologies, could provide valuable improvements for both clinical and home therapies and monitoring. In the clinics, or in little rehabilitation centers and laboratories, it could support clinical scales evaluation, providing low-cost and timesaving motor assessments. At home, during the execution of unsupervised domestic training, or during daily-life activities, the low-cost technology, coupled with biomechanical evaluation modules could be helpful for setting training difficulty, when integrated into virtual applications, giving feedback to the patients for motivation and monitoring the quality of training and life at distance.

Supported by promising evidences found in the literature, especially when considering healthy people, in this paper Kinect V2 is presented as a tool for the evaluation of the motor performances of neurological patients, during the execution of the Reaching Movement (RM), further exploiting the data presented in previous studies [50]. Thus, this paper investigates and characterizes the presence of differences in performances of healthy subjects, during the execution of RM, compared to post-stroke hemiparetic patients, with a more or a less-impaired limb, with the aim of better understanding the possibility of building strong rehabilitation paradigms, based on low-cost tracking technologies.

## 2. Materials and Methods

### 2.1. Aim

The aim of the study was to investigate the biomechanical performances of healthy subjects and neurological hemiparetic patients in RM movements, with the Kinect V2 commercial, low-cost sensor, and test whether the sensor is able to discriminate differences between pathology and healthiness.

### 2.2. Settings

The experiment on healthy people was conducted at the Institute of Intelligent Industrial Systems and Technologies for Advanced Manufacturing (STIIMA) of the Consiglio Nazionale delle Ricerche (CNR), Lecco and Milano, Italy. Recruitment of participants took place at the Institute of Intelligent Industrial Systems and Technologies for Advanced Manufacturing (STIIMA) of the Consiglio Nazionale delle Ricerche (CNR), Milano, Italy. The experiment on the patients was conducted at the Villa Beretta Rehabilitation Hospital (Costa Masnaga, Italy).

### 2.3. Participants

For healthy people, criteria for eligibility were—being neurologically and orthopedically intact [50]. A cohort composed of 77 healthy subjects (46 males, 31 females, mean age 41.87 ± 19.34), unaware of the purpose of the study, was enrolled for the experiment, after giving an informed consent. The characteristics of the subjects are summarized in Table 1. Patients were in the late subacute or chronic phase of the disease (>5 months after the stroke); recruitment criteria included a correct understanding of the task to perform. Their characteristics are summarized in Table 1 and Table 2. Written informed consent was obtained from each subject, before inclusion in the study. The study was reviewed and approved by the CNR Ethics: Commissione per l’etica della Ricerca e la Bioetica del CNR, and the local Ethics Committee at A. Manzoni Hospital, Lecco, and was conducted in compliance with the Declaration of Helsinki. The protocol approval number was 0044338/2018. For the patients, the criteria of eligibility were not imposed on the impaired limb functionality, while capability of understanding the task to be performed was requested.

### 2.4. Experimental Set-up

**Figure 1:**
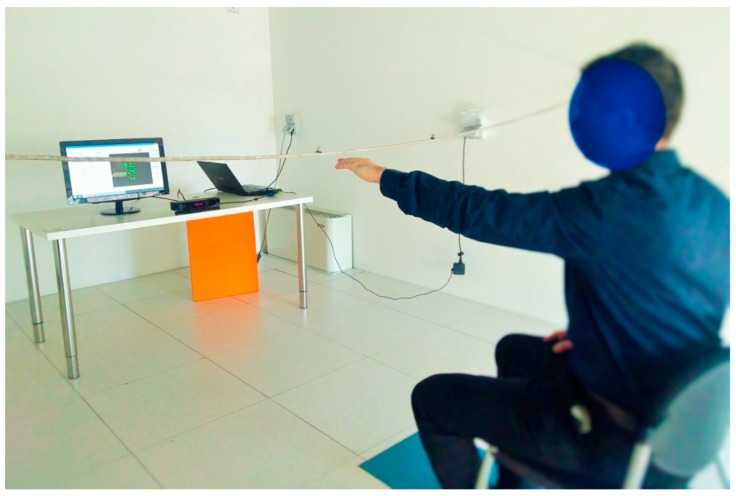
The set-up for Kinect recordings, portrayed in the movement analysis room in Consiglio Nazionale delle Ricerche (CNR), Systems and Technologies for Advanced Manufacturing (STIIMA) Lecco, replicated in CNR–STIIMA Milano, and in the Villa Beretta Rehabilitation Hospital.

During the trials, the experimental set-up (Figure 1) was composed of:

- A Microsoft Kinect V2 sensor version 2.0, mounted on an easel and placed at about a 2.0 m distance from the torso of the subject. The 2.0 m distance was chosen since it is halfway from 0.8 and 3.2 m, which were indicated by Microsoft as the range of distances to use for exploiting the tracking functions of the device. The Kinect V2 was placed about 10 cm under shoulder height, and precisely in front of the subject, about halfway between the limbs. A tolerance of some centimeters had to be accepted, due to the patients’ different anthropometry and sitting postures. Considering that the use of Kinect V2 was meant for future use, even in environments that are non-supervised by medical personnel (such as the patient’s home), where even less-controlled conditions are found, this approximations were considered as reasonable.
- In house software C# for on-line feedback and data logging;
- A PC with Microsoft Windows 8.1, USB 3.0, and Microsoft Kinect One S.D.K. version 2.0;
- A screen providing on-line visual feedback to the operator to visually monitor the correctness of the kinematic tracking.

**Figure 2:**
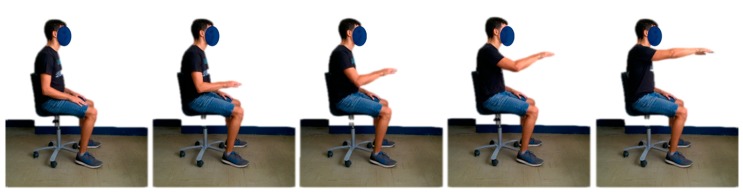
The reaching movement (RM).

The enrolled subjects performed the RM (portrayed in Figure 2), according to the protocol described in [50]. The RM was chosen as it is a paradigmatic gesture for upper-limb rehabilitation, and is fundamental for autonomy in daily life activities, because it simultaneously: (1) involves a multi-joint coordination, (2) involves the capability of elevating the arm against gravity, (3) allows to reach for desired objects, (4) allows interaction with the environment. The coordinated capability of performing RM, allows a wide exploration of the workspace of the upper-limb and a purposeful interaction with the environment. Such choice of motor task also stresses the capability of moving against gravity, which is one of the capabilities that might strongly affect the quality of life of post-stroke patients.

**Figure 3:**
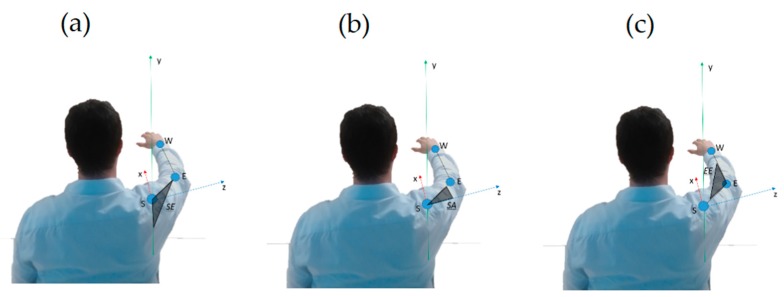
(**a**) The Shoulder Elevation (SE) angle is highlighted. When the limb is leaning along the body, the chosen convention is SE = 0°. When the limb is elevated at shoulder height, as approximately in the figure, SE = 90°. (**b**) The Shoulder Rotation along the Vertical Axis (SA) angle is highlighted. When the limb is aligned on the line that links the shoulders, the chosen convention is SA = 0°. When the limb is frontally positioned (or elevated) at shoulder height, as approximately in the figure, SA = 90°. (**c**) The Elbow Extension angle (EE) is highlighted. When the forearm is completely flexed (arm and forearm aligned, and oriented inversely) the chosen convention is SE = 180° (human beings cannot reach such value). When the limb is fully extended, as approximately in the figure, EE = 0°.

Subjects were sitting comfortably on a chair, with their back straight. They were asked not to move their torso, during the experiment, and to perform the motor tasks, only by limb motions. The considered motor gestures are shown in Figure 3. A target for the RM was set at shoulder height, indicating the point toward which subjects had to point. Subjects were requested to perform the repetitions of the gestures at a natural, comfortable, self-selected speed, with no pauses between one repetition and the following. The starting position was with the elbow flexed, at about 90°, with a pronated hand leaning on the thigh. Each subject performed two acquisitions—RM performed with their dominant and non-dominant limbs. Twelve repetitions of the motor task were performed per acquisition.

The Microsoft Kinect V2 sensor was used to record the movement execution. In total, 77 healthy subjects and 20 hemiparetic patients executed two acquisitions, for a total of 154 records on healthy people and 40 records on patients.

### 2.5. Patient Clinical Assessment: The Reaching Performance Scale

A physicist evaluated each patient with the Reaching Performance Scale [49,51], which is a recently developed assessment used for considering the main motor capabilities involved in the reaching gesture.

### 2.6. Data Sources and Measurements

Tracking data acquired with the Kinect V2 were recorded and logged with an in-house C# software developed for visual feedback and data acquisition. Offline analysis was performed by the means of an in-house developed Matlab software.

3D joints tracking data of shoulder, elbow, wrist, and time labels were recorded and logged for off-line analysis. In order to eliminate noise, the tracking data were low-pass Butterworth filtered, third-order, with a cut-off frequency of 6 Hz. An algorithm for automatic phase detection was implemented. It was needed to separate the forward phase of the movements from the backwards one. All outcome measures were performed in the forward phase.

### 2.7. Outcome Measures

The recorded data were used to compute the following outcome measures (according to the revision of a previously presented protocol [50]):

#### 2.7.1. Kinematics and Range of Motion

- Shoulder Elevation angle (SE, °)
- Shoulder Rotation along Vertical Axis angle (SA, °)
- Elbow Extension angle (EE, °)

Referring to Figure 3, Shoulder Elevation angle is defined as:

$$SE=a\cos(-{\vec{u}}_{y}\cdot {\vec{u}}_{SE})$$

Shoulder Rotation along Vertical Axis angle is defined as:

$$SA=a\cos({\vec{u}}_{z}\cdot {\vec{u}}_{SE})$$

Elbow Extension angle is defined as:

$$EE=a\cos({\vec{u}}_{SE}\cdot {\vec{u}}_{EW})$$

For the conventions adopted, see Figure 3.

The above listed parameters accounted for the movement kinematics (range of motion) at the end of the forward phase of the movement.

#### 2.7.2. Compensatory Strategies

- Trunk Compensus (TC, [])
- Scapular Elevation (ScE, m)

The parameters listed above account for the conventional motor strategies used by patients, to compensate some of their motor impairments. The trunk compensus is a normalized parameter that takes into account the amount of movement, produced by displacing the trunk, rather than by moving the hand towards the target. Defining the trunk displacement from the origin position as OT, and the total displacement of the hand (from begin to end position) as OH, TC is defined as follows:

TC=max(OT)/(OH−max(OT))

If the trunk is not displaced, OT ➔ 0, while, for a “virtual movement” done only with the trunk compensus, OT ➔ 1. In neurological patients, TC might be increased with respect to healthy people, since trunk compensus strategies are used. 

Scapular elevation is measured in meters (m), and addresses how much the shoulder is translated, vertically, to achieve the target. In neurological patients, scapular elevation tends to be increased with respect to healthy people.

#### 2.7.3. Motor Control and Motion Quality

Average number of velocity peaks (VP), used as a measure of smoothness.

The above listed parameter accounted for the movement’s quality of execution, representing smoothness. It is well-known that bell-shaped velocity profiles (single peak) are usually employed in such reaching movements [49]; the more velocity peaks are found, the more jerky is the movement. Thus, the number of velocity peaks is a measure of the movement smoothness.

The protocol was designed to be a simplified, synthetic version, made of a selection of the parameters computed in the clinical environments, during robotic therapies [51].

The present study aims at analyzing the influence of limb dominancy, gender and age, on the biomechanics of daily-life gestures, when measured with a Kinect V2 sensor.

### 2.8. Study Design

In this work, four main experimental cases were considered: (1) Healthy Dominant Limb test; (2) Healthy non-Dominant Limb test; (3) Patients with Impaired Limb test; (4) Patients with Less-Impaired Limb test. Each subset was tested in comparison to the others, on each variable of the biomechanical assessment. Means and standard deviations were computed along with *p*-values of the comparisons, for a total of six multi-group comparisons, one for each of the biomechanical parameters.

### 2.9. Statistics

In the main investigation, for each dependent variable belonging to the evaluation protocol and to a specific data subset to be tested, the normality of the distribution was assessed using the Kolmogorov–Smirnov normality test. Normality and statistical tests were performed with Matlab 2018a. For testing the biomechanical differences on each of the six measures used in the assessment, the one-way ANOVA test was used. For each of the measures, four groups were tested—healthy dominant limb, healthy non-dominant limb, patients with impaired limb, and patients with less-impaired limb. In case biomechanical differences between groups were found, Post-hoc tests (Matlab multcompare) were conducted to understand which group(s) differed from the others. The alpha-error significance level was set to 0.05, for all tests.

## 3. Results

All patients could perform the complete clinical trial, each one according to his motor functionality.

All the scores for the Reaching Performance Scale (RPS) are reported in Table 3. The population is quite heterogeneous, with RPS scores ranging from (0/18) to (18/18), mean 10.6, std 5.1. For a detailed analysis of the correlation between the observed range of motion at shoulder and elbow level and the RPS scores, please see a previous work by the authors’ research group [49]. 

**Figure 4:**
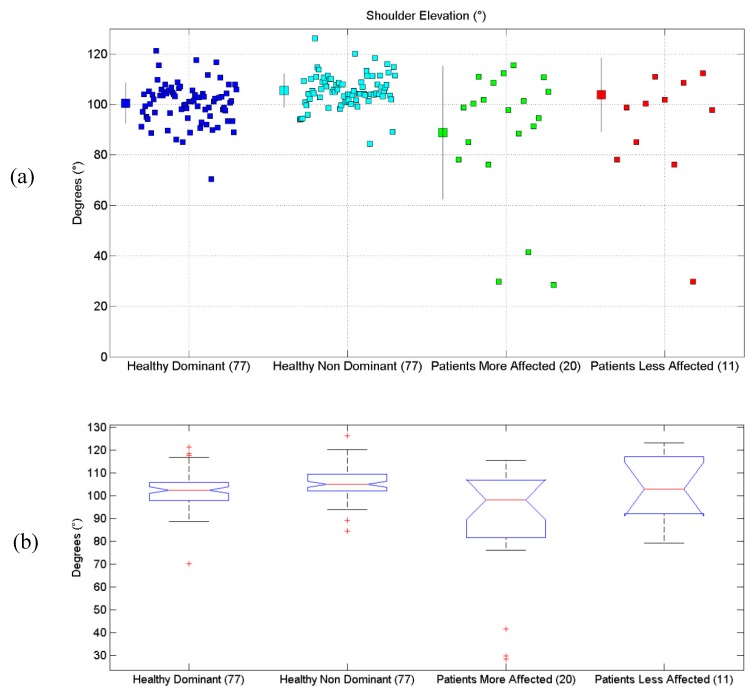
(**a**) Scatterplot for Shoulder Elevation Angle for the four considered groups. Mean (SD) of each population (considered limb), are reported. (**b**) Distributions of Shoulder Elevation Angle, are shown for the four experimental conditions. The central line indicates the median, while the lower and upper extremities of the boxes represent the 25th and 75th percentiles, respectively. The whiskers comprise all the data points distribution, except the outliers, which are plotted separately, using the ‘+’ mark.

**Figure 5:**
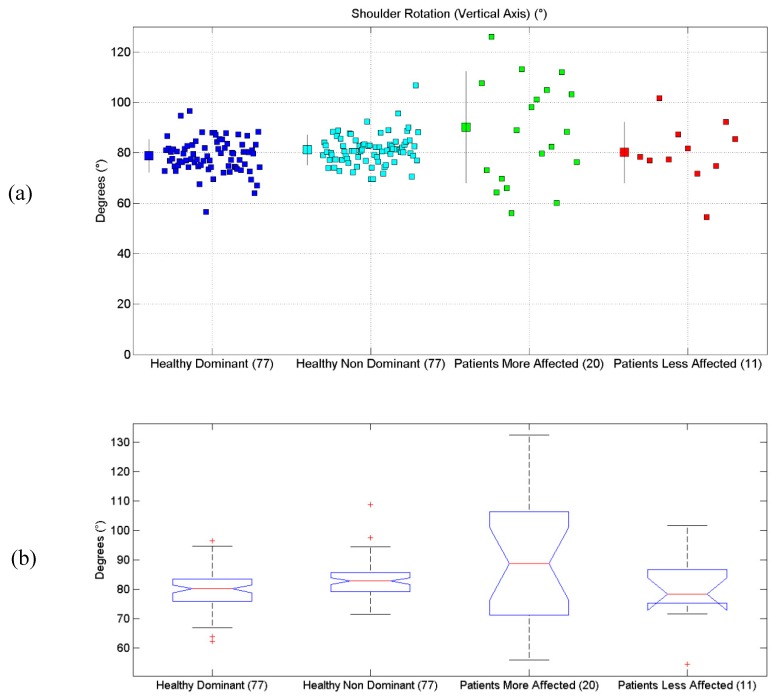
(**a**) Scatterplot for Shoulder Rotation Angle for the four considered groups. Mean (SD) of each population (considered limb) are reported. (**b**) Distributions of Shoulder Rotation Angle, are shown for the four experimental conditions. The central line indicates the median, while the lower and upper extremities of the boxes represent the 25th and 75th percentiles, respectively. The whiskers comprise all the data points distribution, except the outliers, which are plotted separately using the ‘+’ mark.

**Figure 6:**
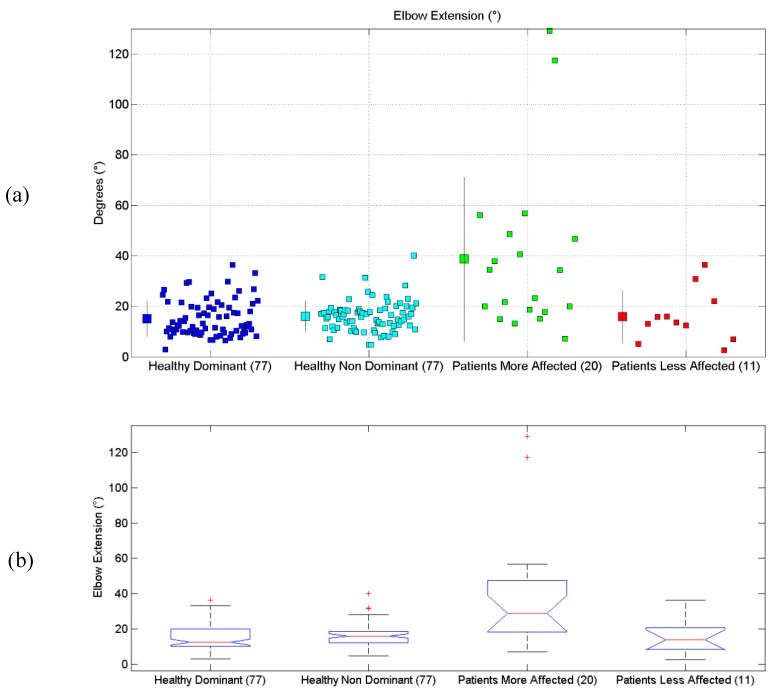
(**a**) Scatterplot for Elbow Extension Angle for the four considered groups. Mean (SD) of each population (considered limb) are reported. (**b**) Distributions of Elbow Extension Angle, are shown for the four experimental conditions. The central line indicates the median, while the lower and upper extremities of the boxes represent the 25th and 75th percentiles, respectively. The whiskers comprise all the data points distribution except the outliers, which are plotted separately using the ‘+’ mark.

**Figure 7:**
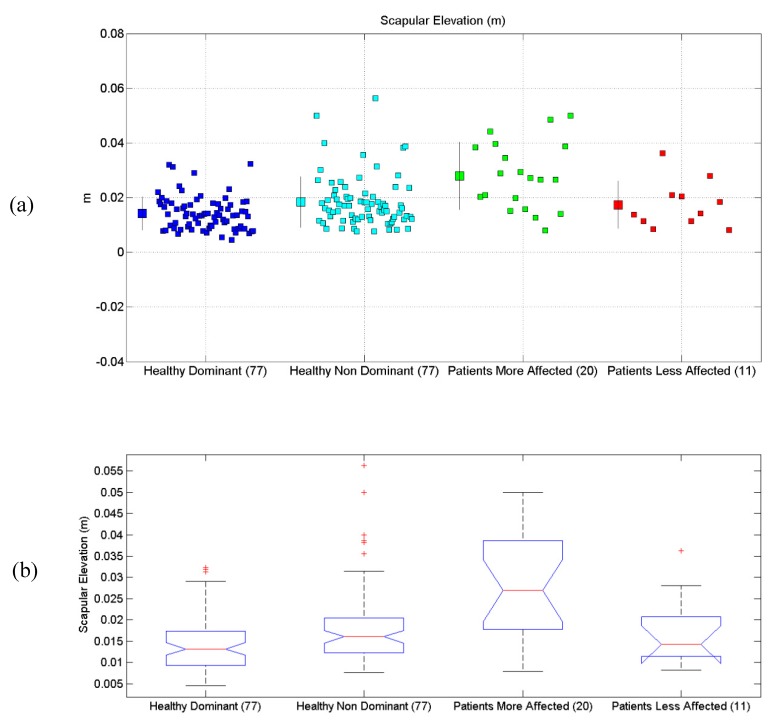
(**a**) Scatterplot for Scapular Elevation for the four considered groups. Mean (SD) of each population (considered limb) are reported. (**b**) Distributions of Scapular Elevation, are shown for the four experimental conditions. The central line indicates the median, while the lower and upper extremities of the boxes represent the 25th and 75th percentiles, respectively. The whiskers comprise all the data points distribution except the outliers, which are plotted separately using the ‘+’ mark.

**Figure 8:**
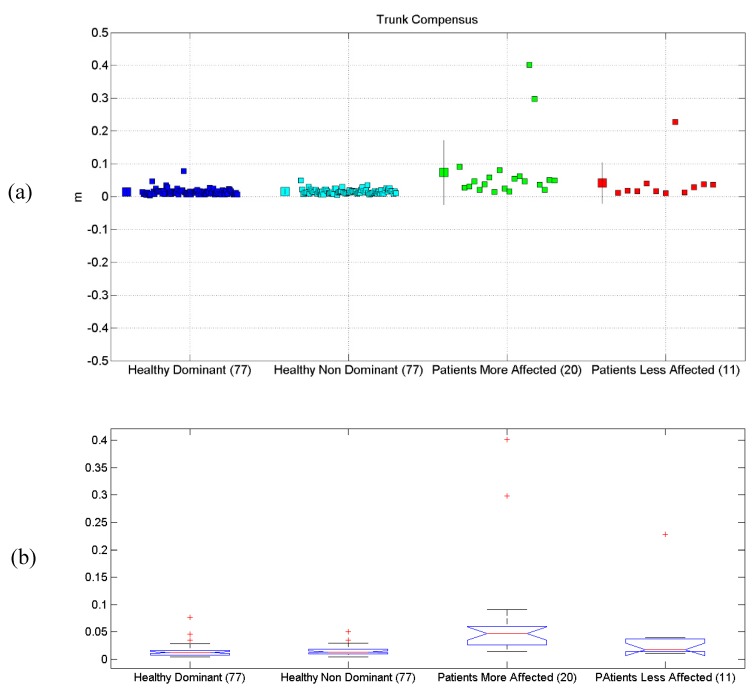
(**a**) Scatterplot for Trunk Compensus for the four considered groups. Mean (SD) of each population (considered limb) are reported. (**b**) Distributions of Trunk Compensus, are shown for the four experimental conditions. The central line indicates the median, while the lower and upper extremities of the boxes represent the 25th and 75th percentiles, respectively. The whiskers comprise all data points of distribution, except the outliers, which are plotted separately using the ‘+’ mark.

**Figure 9:**
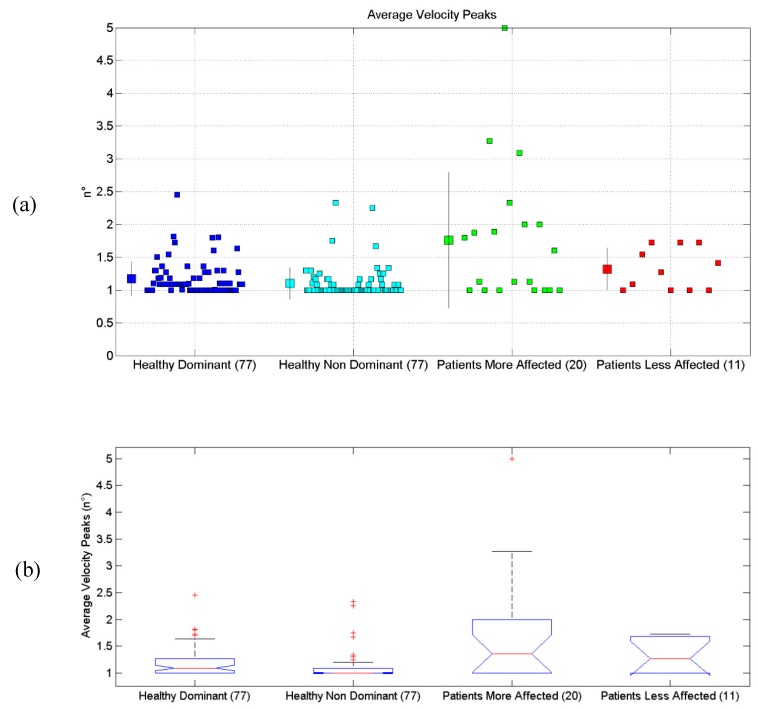
(**a**) Scatterplot for the Average Number of Velocity Peaks for the four considered groups. Mean (SD) of each population (considered limb) are reported. (**b**) Distributions of the Average Number of Velocity Peaks, are shown for the four experimental conditions. The central line indicates the median, while the lower and upper extremities of the boxes represent the 25th and 75th percentiles, respectively. The whiskers comprise all data points of distribution except the outliers, which are plotted separately using the ‘+’ mark.

All the results are synthetically reported in Table 4. All scatterplots for each of the six proposed evaluations, for each of the considered limbs, are reported in Figure 4a, Figure 5a, Figure 6a, Figure 7a, Figure 8a and Figure 9a, along with the results of the ANOVA test (Figure 4b, Figure 5b, Figure 6b, Figure 7b, Figure 8b and Figure 9b).

### 3.1. Shoulder Elevation Angle

Results related to Shoulder Elevation Angle are reported. Statistical differences were found between the four groups, according to the ANOVA test (*p* < 10^−6^). Post-hoc tests (Matlab multcompare) revealed that the mean of the Patients More-Affected Limb group was different from one of the other groups.

### 3.2. Shoulder Rotation Angle (Along Vertical Axis)

Results related to Shoulder Rotation Angle are reported. Statistical differences were found between the four groups, according to the ANOVA test (*p* = 0.0004). Post-hoc tests (Matlab multcompare) revealed that the mean of the Patients group was different from the one of the other groups.

### 3.3. Elbow Extension Angle

Results related to Elbow Extension Angle are reported. Statistical differences were found between the four groups, according to the ANOVA test (*p* < 10^−10^). Post-hoc tests (Matlab multcompare) revealed that the mean of the Patients More-Affected Limb group was different from one of the other groups.

### 3.4. Scapular Elevation

Results related to Scapular Elevation are reported. Statistical differences were found between the four groups, according to the ANOVA test (*p* < 10^−10^). Post-hoc tests (Matlab multcompare) revealed that the mean of the Patients More-Affected Limb group was different from one of the other groups.

### 3.5. Trunk Compensus

Results related to Trunk Compensus are reported. Statistical differences were found between the four groups, according to the ANOVA test (*p* < 10^−8^). Post-hoc tests (Matlab multcompare) revealed that the mean of the Patients Less Affected Limb group was different from the one of the other groups. The Patients with the Less-Affected Limb group was also different from the others, probably due to outliers.

### 3.6. Average Number of Velocity Peaks

Results related to the Average Number of Velocity Peaks are reported. Statistical differences were found between the four groups, according to the ANOVA test (*p* < 10^−8^). Post-hoc tests (Matlab multcompare) revealed that the mean of the Patients with More-Affected Limb group was different from the one of the other groups, as well as the Patients with Less-Affected Limb group, which was also different from the others.

## 4. Discussion

### 4.1. On the Biomechanical Results

As expected by previous findings in the literature [50], no observable differences in motor performances were found when considering the healthy dominant and non-dominant limbs in a simple gesture, such as the reaching movement, according to the Kinect V2. It is well-known that dominant and non-dominant limbs have a tendency to specialize in dynamical and static motor tasks respectively [52], and the dominant arm can achieve more varied and flexible control over movement trajectories, while accuracy and precision are comparable [53]. However, such differences would be particularly expected in relation to highly demanding motor tasks (hard to complete or very fast, engaging, not known a-priori) or in fine control (related for example to hand or finger dexterity [54]), “in favor” of the dominant limb, rather than in the daily-life, well-known gestures, such as RM. Considering the quite high number of involved subjects, this result can be considered to be a solid benchmark condition for the assessment of the performances of impaired people. At the same time, this assessment provides further evidence that, at least for the considered simple gesture, the biomechanical performances of the two limbs were comparable. For patients, the assessments provided very different results. In fact, the biomechanical assessment presented in this paper showed, as expected, that in general, the motor performances of a heterogeneous group of patients detracted from the performances of healthy people and of the less-affected limb. This is a relevant result because the “capability of discrimination” of the performances was required to guarantee that Kinect might be a useful device for evaluations in neurorehabilitation. Very interestingly, even if the number of the available subjects was reduced, the patients’ less-impaired limb provided a “halfway performance capability”, which often performed better than the average of the more-affected limb, even if in a worse way, with respect to the healthy benchmark. These results also need to be confirmed on a larger cohort of people. This finding suggests that low-cost tools, such as Kinect might discriminate different level of impairments, as already suggested by previous research [50], and at least identify peculiar features of a group of people or patients (i.e., on average, patients with less-impaired limb show less velocity peaks than the more-affected limb, but both have a more than healthy benchmark).

### 4.2. Kinect in Real Applications

The use of Kinect for rehabilitation has already been tested in the literature, and exploited in several commercial and research applications. In fact, such an approach might substitute, or better integrate, some of the clinical evaluations that are usually administered to patients. A quite detailed overview of the patient’s status can be portrayed and summarized in short reports, highlighting the level of disability with respect to the population of healthy people. Furthermore, this potential can be exploited for the automatization of clinical scales with a quantitative method that does not depend on operator variability, as has already been proposed in previous studies that have tried to perform quantified evaluations of the Fugl-Meyer Assessment, the Reaching Performance Scale, and the Paretic Arm Non-Use assessments [46,47,48,49]. Furthermore, Kinect or equivalent markerless systems could be introduced in clinics and small-scale laboratories, as well as in the home environment, which would increase the possibility of executing tests for the evaluation of motor functionality. The Kinect assessment could also be used for a preliminary, fast evaluation of patients, to direct him to a compatible rehabilitation process, chosen according to his remaining residual functionality.

### 4.3. Limitations

This study has several limitations. First, only the paradigmatic gesture has been considered. The validity of the methodology should be tested on a wider group of motor gestures. Furthermore, while a quite high number of healthy people were involved (77 participants), the cohort of patients was not as large (20 patients), and data from both limbs were available only for a subgroup of patients (11 patients). Thus, while the statistical significance of some results was reported, the statistical power, especially on the sample of patients, was limited. However, some trends and preliminary statistics could be presented. The proposed protocol gave only a summarized overview of the patients’ capabilities, but it still has a large margin for being enriched and improved with further assessments. Last, the authors consider it to be very important to test the presented approach in a real-life scenario, involving clinics, laboratories, and patients’ home.

## 5. Conclusions

In this paper, Kinect V2 was used to test a simple biomechanical protocol on the dominant and non-dominant limbs of healthy people, and on the less and more impaired limbs of post-stroke patients. Preliminary results suggest that healthy dominant and non-dominant limbs show comparable performances, as well as patients’ less affected limb. On the contrary, Kinect V2 and clinical scales detect poorer performances for the more affected limb. Further developments of the concept presented in this paper will include refinement of the biomechanical protocol, and testing in scenarios including patients’ home and clinics. 

## Figures and Tables

**Table 1:** Summary of participant data.

**Males**	**Females**	**Right Dominant**	**Left Dominant**	**Mean Age**	**Total**
46	31	72	6	41.77 ± 19.29	77
**Males**	**Females**	**Right Impairment**	**Left Impairment**	**Mean Age**	**Total**
11	9	12	8	52.21 ± 16.43	20

**Table 2:** Demographic data of patients.

Patient	Gender	Age Range	Impaired Limb
Pt1	Male	70–80	Right
Pt2	Female	60–70	Right
Pt3	Male	50–60	Right
Pt4	Male	50–60	Right
Pt5	Female	30–40	Right
Pt6	Male	40–50	Right
Pt7	Female	60–70	Left
Pt8	Female	20–30	Left
Pt9	Male	60–70	Right
Pt10	Male	60–70	Right
Pt11	Female	20–30	Left
Pt12	Female	20–30	Right
Pt13	Male	60–70	Left
Pt14	Male	50–60	Left
Pt15	Male	50–60	Right
Pt16	Male	60–70	Left
Pt17	Male	60–70	Right
Pt18	Female	40–50	Right
Pt19	Female	40–50	Left
Pt20	Female	70–80	Left

**Table 3:** Summary of the reaching performance scale scores.

Patient ID	Trunk	Smoothness	Shoulder	Elbow	Prehension	Global	Total
Pt1	2	2	2	2	2	2	12
Pt2	3	2	2	3	3	3	16
Pt3	1	1	1	0	1	0	4
Pt4	3	1	3	1	2	2	12
Pt5	3	3	2	3	3	3	17
Pt6	2	2	3	3	0	1	11
Pt7	3	0	3	3	1	2	12
Pt8	3	3	3	1	0	1	11
Pt9	3	3	2	3	3	3	17
Pt10	2	1	1	2	0	1	7
Pt11	0	0	0	0	0	0	0
Pt12	2	2	2	2	0	1	9
Pt13	3	2	1	1	2	1	10
Pt14	3	2	2	2	2	2	13
Pt15	3	0	0	1	0	0	4
Pt16	3	0	0	0	0	0	3
Pt17	3	2	2	2	3	2	14
Pt18	2	3	2	3	3	2	15
Pt19	2	2	1	2	0	1	8
Pt20	3	3	3	3	3	3	18

**Table 4:** Summary of the results of the biomechanical assessment.

Measure	Healthy Dominant (°)	Healthy Non-Dominant (°)	Patients Impaired (°)	Patient Less Impaired (°)	Have Groups Different Mean?
SE	101.9(8.0)	105.6(6.6)	88.8(26.3)	103.8(14.7)	*p* < 10^−6^
SA	79.9(6.3)	81.2(6.0)	90.2(22.2)	80.2(12.1)	*p* = 0.0004
EE	15.3(7.1)	16.1(6.1)	38.7(32.5)	15.9(10.4)	*p* < 10^−10^
ScE	0.0139(0.062)	0.0184(0.093)	0.0280(0.0124)	0.017(0.08)	*p* < 10^−10^
TC	0.0146(0.0302)	0.0157(0.073)	0.0736(0.0981)	0.0418(0.0628)	*p* < 10^−8^
VP	1.1743(0.2624)	1.1037(0.2401)	1.7618(0.0339)	1.3189(0.3197)	*p* < 10^−8^
